# 192-dimensional wavelength-/mode-/polarization-multiplexed photonic architecture for highly parallel optical computing

**DOI:** 10.1093/nsr/nwag350

**Published:** 2026-06-08

**Authors:** Qingrui Yao, Yuxin Sun, Weike Zhao, Yiwei Xie, Yingying Peng, Huan Li, Pan Wang, Xiangxing Bai, Yaocheng Shi, Daoxin Dai

**Affiliations:** State Key Laboratory for Extreme Photonics and Instrumentation, College of Optical Science and Engineering, International Research Center for Advanced Photonics, Zhejiang University, Hangzhou 310058, China; QIANYUAN Laboratory, Hangzhou 310058, China; State Key Laboratory for Extreme Photonics and Instrumentation, College of Optical Science and Engineering, International Research Center for Advanced Photonics, Zhejiang University, Hangzhou 310058, China; State Key Laboratory for Extreme Photonics and Instrumentation, College of Optical Science and Engineering, International Research Center for Advanced Photonics, Zhejiang University, Hangzhou 310058, China; State Key Laboratory for Extreme Photonics and Instrumentation, College of Optical Science and Engineering, International Research Center for Advanced Photonics, Zhejiang University, Hangzhou 310058, China; State Key Laboratory for Extreme Photonics and Instrumentation, College of Optical Science and Engineering, International Research Center for Advanced Photonics, Zhejiang University, Hangzhou 310058, China; State Key Laboratory for Extreme Photonics and Instrumentation, College of Optical Science and Engineering, International Research Center for Advanced Photonics, Zhejiang University, Hangzhou 310058, China; State Key Laboratory for Extreme Photonics and Instrumentation, College of Optical Science and Engineering, International Research Center for Advanced Photonics, Zhejiang University, Hangzhou 310058, China; QIANYUAN Laboratory, Hangzhou 310058, China; State Key Laboratory for Extreme Photonics and Instrumentation, College of Optical Science and Engineering, International Research Center for Advanced Photonics, Zhejiang University, Hangzhou 310058, China; Intelligent Optics and Photonics Research Center, Jiaxing Research Institute, Zhejiang University, Jiaxing 314000, China; Ningbo Research Institute, Zhejiang University, Ningbo 315100, China; State Key Laboratory for Extreme Photonics and Instrumentation, College of Optical Science and Engineering, International Research Center for Advanced Photonics, Zhejiang University, Hangzhou 310058, China; Intelligent Optics and Photonics Research Center, Jiaxing Research Institute, Zhejiang University, Jiaxing 314000, China; Ningbo Research Institute, Zhejiang University, Ningbo 315100, China

**Keywords:** multidimensional multiplexing, reconfigurable optical computing, optical computing, large-scale optical computing, parallel processing, acceleration processing

## Abstract

Photonic matrix–vector multiplication (MVM) constitutes a core computational primitive in on-chip photonic neural networks, enabling ultrahigh-speed multiply–accumulate operations. Most existing photonic MVM implementations do not fully exploit the intrinsic multidimensional degrees of freedom of light, thereby constraining parallelism, scalability, and functional flexibility. Here, we present a 192-dimensional multiplexed photonic computing architecture that simultaneously harnesses 32 wavelengths, three spatial modes, and dual polarizations, enabling highly parallel and fully reconfigurable optical computation within a single integrated platform. By dynamically selecting and combining multiplexed channels, we demonstrate flexible convolution-kernel reconfiguration, supporting large and variable kernel sizes up to 13 × 13, as well as multichannel parallel signal processing. Enabled by large-scale and adaptable convolution operations, we experimentally demonstrate effective suppression of structured noise, enhanced extraction of diverse spatial features, and preservation of holistic contour information—capabilities that are difficult to achieve using conventional small-kernel architectures alone. The optical chip can achieve a throughput of 15.36 tera-operations per second, which can be further improved by adopting higher-speed modulation and expanding the optical dimension channels. Overall, the proposed architecture establishes a powerful and scalable photonic computing paradigm, with strong potential for computer vision, biomedical imaging, and next-generation artificial intelligence systems, especially those demanding high-precision visual processing.

## Teaser

A 192-dimensional multiplexed photonic computing architecture enables fully reconfigurable large-kernel optical convolution for high-precision AI vision tasks.

## INTRODUCTION

Matrix–vector multiplication (MVM) is the core computational primitive of convolution neural networks (CNNs), serving as the building block for high-speed multiply–accumulate operations. In particular, convolution operations in CNNs can be reformulated as a series of MVMs, making MVM efficiency crucial for large-scale neural computing. Electronic architectures favor a ‘small-yet-deep’ paradigm, stacking small kernels (e.g., 3 × 3) to emulate larger receptive fields with fewer parameters and lower complexity [1,2]. This approach, exemplified by VGGNet and ResNet, is well supported by GPUs optimized for massive small-kernel operations [3,4]. However, this paradigm—though highly efficient in electronic computing—faces significant challenges in emerging optical computing architectures, particularly in silicon-photonics platforms [5]. There are several physical constraints, including the cumulative insertion losses as the scale increases [6], lack efficient and low-power optical nonlinear activation mechanisms [7], mismatch between one-dimensional waveguide arrays and two-dimensional image/kernel data formats [8]. These constraints render the ‘small-yet-deep’ strategy used in electronics impractical, while making ‘large-yet-shallow’ architectures with reconfigurable large kernels a more natural and scalable solution [9].

Within this context, developing reconfigurable large-scale optical convolutional kernels is of critical importance. Currently the realization of matrix multiplication mainly relies on multiport interferometric networks [7,10–18] or wavelength-division multiplexing (WDM) techniques [19–22]. Mach–Zehnder interferometer (MZI) meshes enable flexible optical computing by controlling the amplitude and phase of optical signals. Even though the performance of individual MZI units continues to be enhanced through optimal designs [23], the system scaling remains severely limited due to the inevitably increased insertion losses and calibration complexity. Generally, scaling beyond 16 channels remains challenging [12,22,24,25]. Using WDM with multiple wavelength channels carrying different data streams enables parallel multiply–accumulate operations [26–28]. However, the fundamental limit for scalability includes the limited free spectral range (FSR) of wavelength-selective photonic devices. Leveraging multiple optical degrees of freedom (DoFs) of the lightwave therefore provides a promising routine toward large-scale optical computation. Recently WDM-compatible optical mode division multiplexing neural network accelerator was explored by multiplexing multiple wavelengths and multiple modes [29]. However, the optical DoFs are difficult to decouple, and their potential for high parallelism and computational capacity remains underutilized [30–32]. Moreover, because optoelectronic conversions are still required, the associated processing overheads hinder the practical deployment of optical computing systems [33]. Nevertheless, large kernels with reconfigurable sizes offer clear benefits such as expanding the receptive field and enhancing expressiveness [34–36]. This capability is especially valuable in latency- and power-sensitive scenarios such as edge computing [36–38] and is key to advancing optical computing toward general, efficient, and high-performance paradigms [39].

In this work, we propose for the first time a wavelength-, mode-, and polarization-(WMP) multiplexed architecture that intelligently leverages multiple optical DoFs to enable large-scale and flexible convolutional operations. The architecture supports 192 channels (including 32 wavelengths, three modes, and dual polarizations) for parallel computing and the convolution size is up to 13 × 13, with flexible adjustment through dynamic channel selection for a wide range of parallel applications. In particular, elliptic microring resonators (EMRs) with large FSR, along with wavelength-insensitive polarization-/mode-handling devices based on asymmetrical directional couplers, are designed to ensure independent control of each DoF of optical signals. We experimentally demonstrate successful multiscale convolution on images, highlighting how different kernel sizes extract complementary features, and validate the approach on noisy datasets using convolutional neural networks. The proposed architecture can achieve a throughput of 15.36 tera-operations per second (TOPS) and is highly scalable, enabling the incorporation of additional optical dimensions and higher-speed modulation to further enhance chip throughput. These strategies provide a pathway for high-precision large-scale optical computing, with promising applications in computer vision, artificial intelligence, and real-time image processing, and offer a scalable foundation for future multidimensional photonic computing systems.

## DESIGN AND PRINCIPLE

Figure 1a shows the proposed WMP-multiplexed photonic architecture for versatile convolution operations by fully exploiting the available DoF of light, thus greatly enhancing the parallel computing capacity. Here there are three modes, dual polarizations and 32 wavelengths involved to enable a total of 192 channels for parallel computing. The configuration incorporates a six-channel dual-polarization mode demultiplexer with a 6×32 array of EMRs. A multiwavelength optical source is first demultiplexed by a wavelength demultiplexer. Each wavelength channel is then evenly split into six paths (corresponding to three spatial modes and two polarizations), with each path modulated by its respective data stream. The modulated signals are subsequently recombined through mode/polarization combiners and wavelength multiplexers, and then injected into the on-chip WMP-multiplexed photonic computing processor chip for computation. Within the computing processor, the modulated signals carried by the multimode waveguide are demultiplexed into single-mode channels (CH_1_–CH_6_). In each channel, different wavelengths are individually weighted by their corresponding EMRs. Each EMR, equipped with a micro-heater for thermally tuning, is individually controlled to implement the desired weight for the target wavelength-channel. The convolution results corresponding to different processing operations are obtained via selective collection from the designated channels. By selectively combining the signals at the output of each channel (CH_1_–CH_6_), the system enables flexible kernel-size configuration and adjustable parallel processing capability, featuring the function flexibility and reconfigurability. With such a configuration, it is possible to achieve several types of representative parallel processing with (1) an ultra-large-scale convolution operation up to 13 × 13 kernels with 192 elements; (2) different matrix computing across different modes and polarizations for the same input image; (3) different input images and kernels across distinct modes, wavelengths, and polarizations. A schematic illustration of these functionalities is presented in Fig. 1b–d. All functions are implemented on the same chip, with reconfiguration achieved by adjusting the input modulation and subsequent signal processing. For example, in Fig. 1b, ultra-large-scale convolution is realized by loading identical input data onto all modes; after wavelength-dependent weighting via the 169 (13 × 13) EMR array, the outputs from CH_1_–CH_6_ are combined to form the final result. In contrast, for parallel processing, as shown in Fig. 1c and d, different input data are assigned to distinct modes, with each channel processed independently and detected separately, enabling multi-channel parallel computation. Owing to the versatility of the architecture, not all channels are required for every task. Depending on the kernel size, different numbers of modes and wavelengths can be employed, allowing flexible selection of the active modes, wavelengths, and channels.

**Figure 1.**
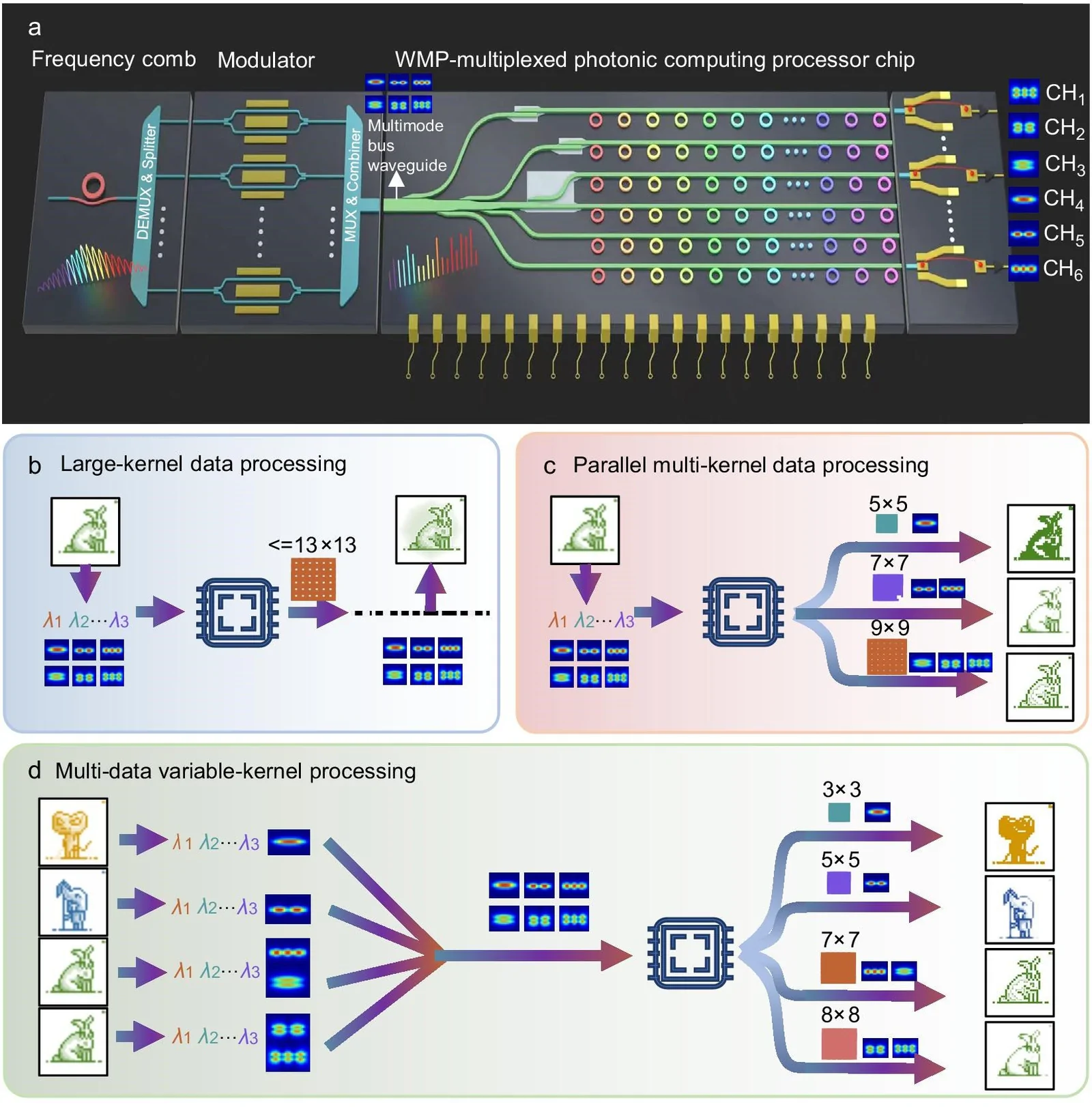
Schematic of the WMP-multiplexed photonic architecture for versatile convolution operations. (a) Schematic of the the proposed WMP-multiplexed photonic architecture for versatile convolution operations. The labeled WMP-multiplexed photonic computing processor chip is the part that is implemented and demonstrated in the silicon chip. DEMUX: demultiplexing; PD: photodetector. The working priciple of each operation: (b) large-scale convolution capability (e.g. 13 × 13 kernels); (c) parallel data processing with each processed with different kernels; (d) independent data modulation and computation with variable kernels.

## EXPERIMENTS AND CHARACTERIZATIONS

The WMP-multiplexed photonic computing processor in Fig. 1a was fabricated on a silicon platform, as shown in Fig. 2a, and the packaged chip is shown in Fig. 2b. The six-channel hybrid mode/polarization (de)multiplexer supports three transverse electric modes (i.e. TE₀, TE₁, and TE₂) and three transverse magnetic modes (i.e. TM₀, TM₁, and TM₂). As indicated in the schematic, the mode conversion and multiplexing functionalities of the ‘MUX & Combiner’ module in Fig. 1a are also integrated on the processor chip. In this configuration, the processor chip inputs are implemented as single-mode ports, each supporting two polarizations. After the dual-polarization modulated signals (each with 32 wavelengths) are injected into the chip, they are combined into multimode signals via on-chip mode MUXs and propagated along a multimode bus waveguide. The signal is subsequently demultiplexed into six TE₀ modes (corresponding to six channels) for signal processing. Details of the chip design and mode conversion are given in Notes S1 and S2, respectively. These signals are then routed through a 6 × 32 array of EMRs for weight loading and convolutional multiplication operations.

**Figure 2.**
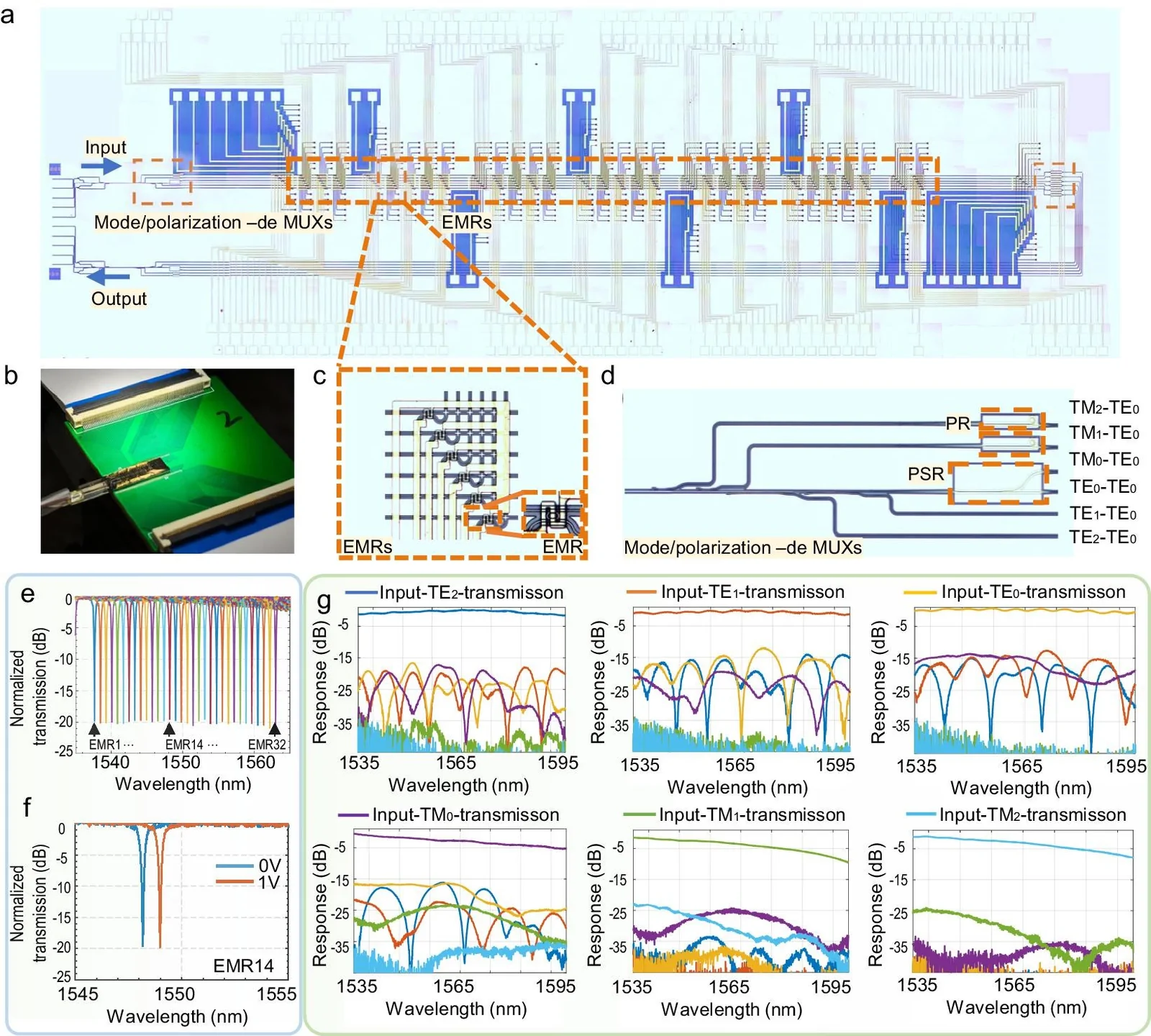
Chip implementation and characterization. (a) Microscope image of the chip. (b) Packaged chip. (c, d) Microscope images of EMRs (c) and MDM-PDM (de)MUXs (d). (e) Transmissions of the EMR array. (f) Transmissions of a single EMR with an applied voltage of 0 and 1.0 V. (g) Transmissions of the fabricated MDM-PDM (de)MUXs.

Figure 2c shows an optical microscope image of the add-drop EMR array, with an inset providing a magnified view of an individual EMR. In the experiments, we employ the through ports for signal transmission, whereas the drop ports enable monitoring of the EMRs, resonance frequency and status, thereby enhancing operational convenience. The EMRs with adiabatically varied radii/widths are designed to shrink the cavity length and minimize the loss. This design enables EMRs to implement a broad bandwidth and a large FSR, supporting a great number of wavelength channels in WDM systems. The details of the EMR design are in Note S2. Figure 2e illustrates the measured through spectral responses of the EMR array, showing that the resonance wavelengths have a channel spacing of 100 GHz with a low loss around 1 dB. Figure 2f depicts the spectral response of a single EMR with thermal-tuning by introducing a micro-heater under a 1-V bias voltage, demonstrating an extinction ratio of ∼20 dB for the signal amplitude control. This efficient modulation capability under a low driving voltage highlights the energy-efficient tuning mechanism for weighting operations in the present EMRs.

Figure 2d shows the microscope image of the dual-polarization mode (de)multiplexer. It incorporates two polarization rotators (PRs) and one polarization splitter-rotator (PSR). Figure 2g presents the measured spectral response of the fabricated mode (de)multiplexer, showing that the inter-mode crosstalk is about –15 dB. The excess loss for the TE modes is less than 2 dB across the 1530–1600 nm wavelength range, while the TM-mode channels have an excess loss of <5 dB in the wavelength range from 1530 to 1585 nm. Further details about the structural design of the dual-polarization mode (de)multiplexer are included in Note S2.

## PARALLEL COMPUTATION OF MULTIFUNCTIONAL CONVOLUTION OPERATIONS

To demonstrate the multifunctional capability of our WMP-multiplexed matrix multiplication chip and the influence of different kernel sizes, we select an image with clear contours and detailed background features for convolutional processing. First, parallel convolution computation was performed for a given image (see Fig. 3b) using three distinct 3 × 3 kernels (*k*_1_, *k*_2_, *k*_3_). In this case, the optical signals from nine wavelength lasers are modulated to input information, then multiplexed together, and subsequently coupled onto the chip. Within the chip, these wavelengths were routed into three channels (CH_4_, CH_5_, and CH_6_) corresponding to three modes, with weight adjustment implemented by the EMRs in each channel. The output of each channel (mode) yielded the result of one convolution, allowing the simultaneous acquisition of all three feature maps. Figure 3a depicts the experimental setup for parallel convolution with three distinct 3 × 3 kernels. The selected kernels (*k*_1_, *k*_2_, *k*_3_) were designed for multidirectional edge detection, bottom-edge detection, and inverted averaging, respectively, and the outputs (Fig. 3c–e) show clear agreement with their target functions as desired.

**Figure 3.**
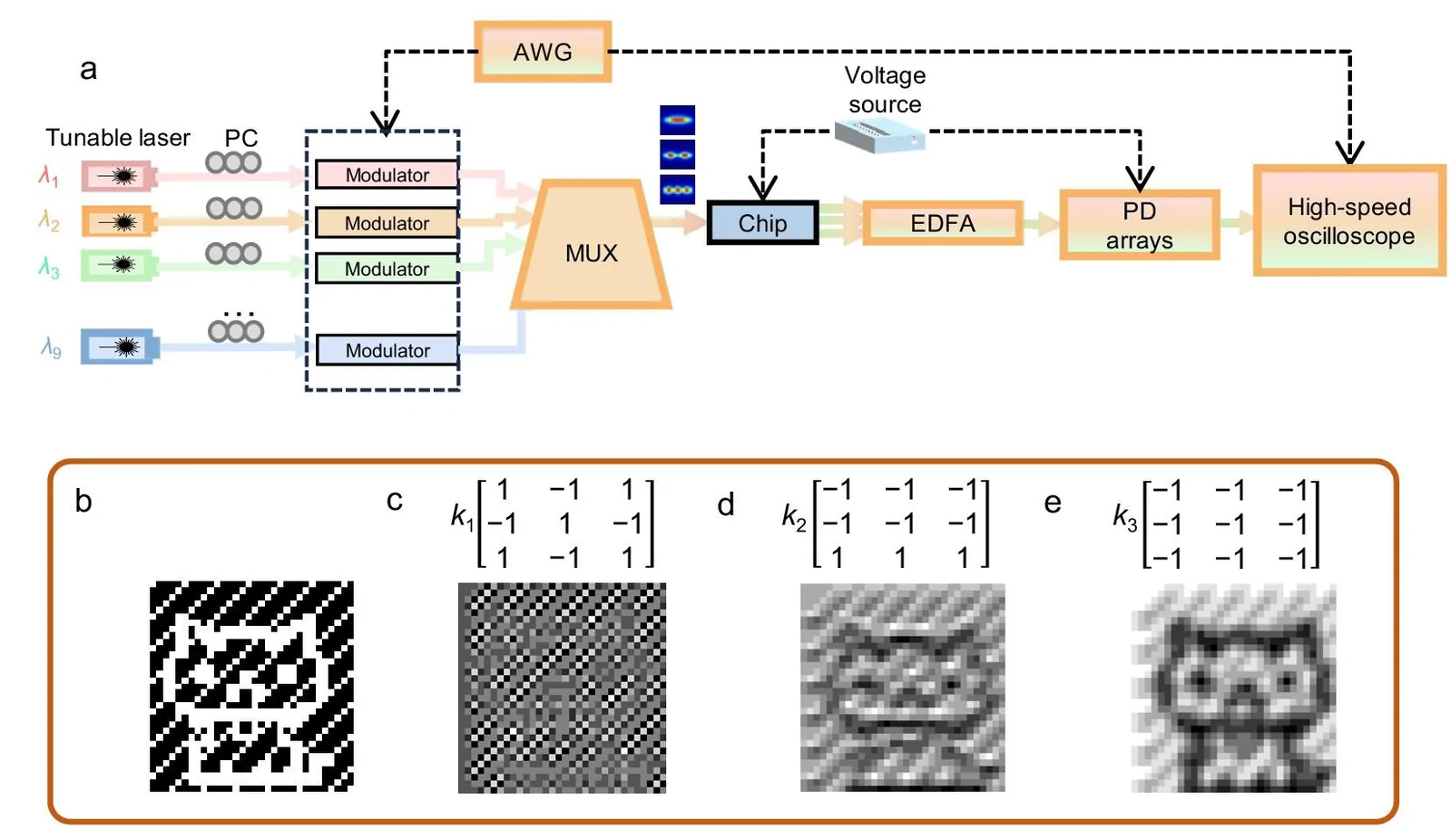
Parallel convolutional processing using three distinct 3 × 3 kernels via wavelength and mode multiplexing. (a) Experimental setup. (b) Input image. (c–e) Output feature maps obtained using kernels *k*_1_ (multi-directional edge detection), *k*_2_ (bottom-edge detection), and *k*_3_ (inverted averaging), respectively. The same input data was modulated simultaneously onto nine wavelengths (*λ*_1_–*λ*_9_) and processed through three spatial modes (TE₀, TE₁, TE₂), enabling concurrent convolution operations with all three kernels. PC, polarization controller; MUX, multiplexer; PD, photodetector.

Subsequently, we applied convolutional operations with different kernel sizes to the same input image as in Fig. 3b. Two representative image-processing functions were demonstrated, including an edge detection filter (EDF, *k*_4_, *k*_5_, *k*_6_) and a mean filter (MF, *k*_7_, *k*_8_, *k*_9_). For each function, three kernel sizes were selected: 3 × 3 (*k*_4_, *k*_7_), 5 × 5 (*k*_5_, *k*_8_), and 7 × 7 (*k*_6_, *k*_9_). These specific kernels used here are depicted in Fig. 4b and e. In the data preprocessing stage, we employed suitable zero-padding to ensure that the output image sizes remained consistent after processing with convolutional kernels of varying dimensions.

**Figure 4.**
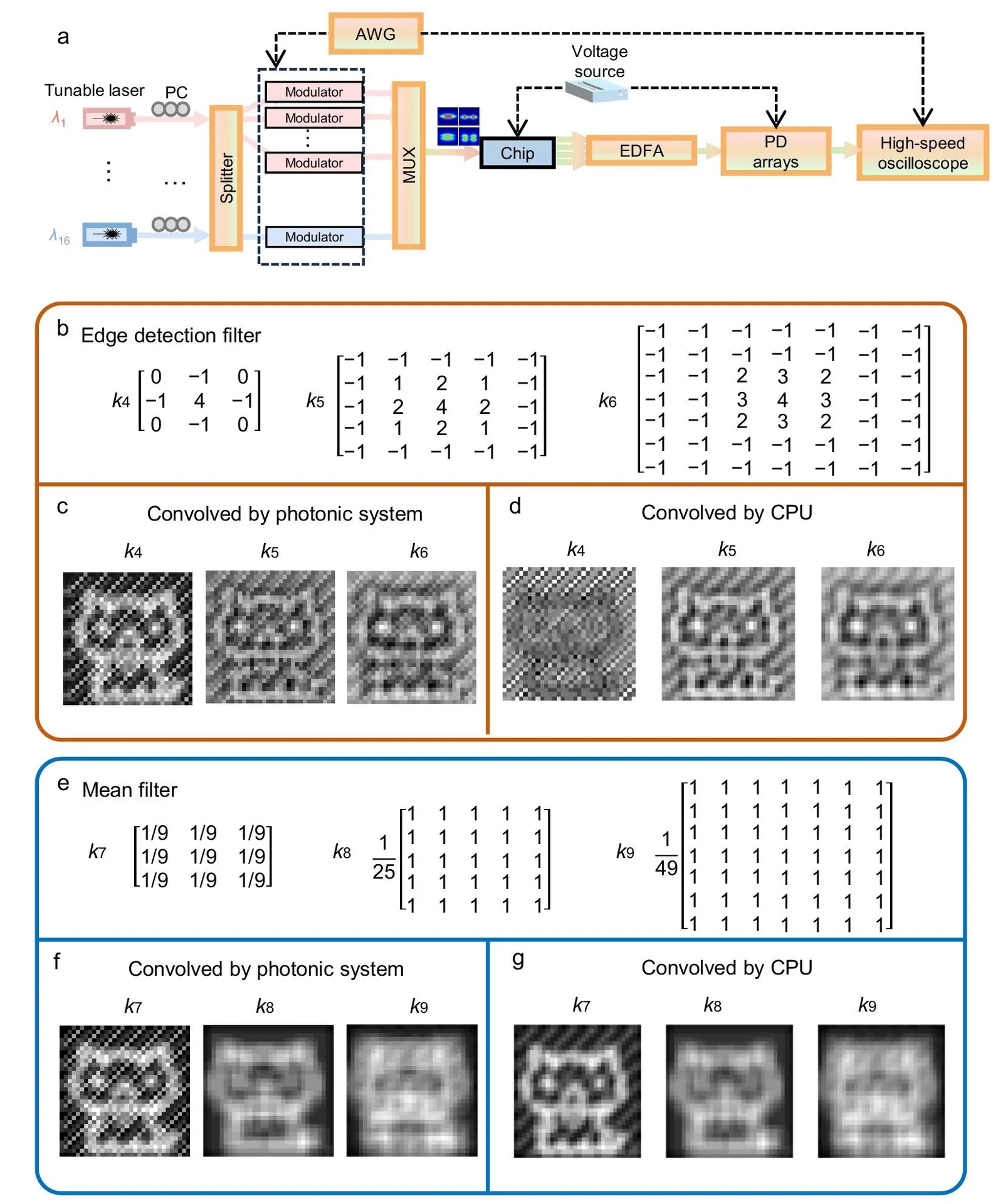
Parallel convolution processing using three different kernel sizes, achieved via wavelength and mode multiplexing. (a) Experimental setup. (b) Parameters of the edge detection filter kernels (*k*₄, *k*₅, *k*₆) with different sizes. (c) Convolution results implemented on the photonic chip using kernels *k*₄, *k*₅, and *k*₆, respectively. (d) Comparison with the corresponding results computed numerically via software. (e) Parameters of the mean filter kernels (*k*₇, *k*₈, *k*₉) with different sizes. (f) Convolution results obtained from the photonic chip using kernels *k*₇, *k*₈, and *k*₉. (g) Comparison with the software-simulated results using the same kernels.

Leveraging the large-scale implementation of the chip, all three convolutions can be executed simultaneously, as shown in Fig. 4a. For the 3 × 3 kernel, nine wavelengths in the TE_0_ mode were used, and the corresponding nine EMRs in CH_4_ are thermally tuned to realize the required weights. The 5 × 5 kernel contains 25 elements, which are mapped onto 25 wavelengths carried by the TE₁ mode. The 7 × 7 kernel consists of 49 elements and therefore requires two optical modes (TM₀ and TM₁), as each mode supports up to 32 wavelengths only. The convolution results for each kernel are obtained by selectively summing the output powers from the designated channels. The processing results for EDF and MF using these three kernel sizes are presented in Fig. 4c and f, respectively, with the corresponding CPU-based reference results presented in Fig. 4d and g. The close agreement between the hardware outputs and software benchmarks validates the reliability of our approach. Details of the image preprocessing and operational procedures are given in Note S3. As illustrated in Fig. 4, larger kernels capture broader structural contours, while smaller kernels preserve fine textural details, reflecting their differential sensitivity to global versus local features.

The distinct receptive fields of kernels of varying sizes generate complementary feature representations, underscoring the importance of multiscale convolutional support. This capability is particularly critical in fields such as medical imaging, where large kernels help delineate organ boundaries and spatial context, whereas small kernels detect localized fine structures such as micro-calcifications or subtle fractures. The reconfigurable optical kernels offer practical value by enabling dynamic multiscale feature extraction on a single platform, eliminating the need for complex physical stacking or repeated optoelectronic conversions. This flexibility further facilitates the implementation of modern large-kernel vision models, improving the efficiency of complex visual processing tasks.

In this work, convolution operations were performed at a data rate of 40 Gbaud, and the results were captured using a high-speed oscilloscope. The computational throughput of the photonic chip is derived from the combination of two polarizations, six modes, and 32 wavelength channels, yielding a total of 15.36 TOPS, calculated as 2 × 6 × 32 × 40 Gbaud. Due to the limited channel count of the arbitrary waveform generator (AWG), multiple experimental runs are required when a larger number of channels are needed. Given a sufficient number of AWGs, our architecture can support multichannel parallel operation. A comprehensive performance characterization, including throughput, computational density, and energy efficiency, is provided in Note S4.

## DENOISING WITH VARIED CONVOLUTIONAL KERNELS

To validate the computational capability of our system and the importance of multiscale kernels, we conducted image denoising experiments using convolutional operations with different kernel sizes. A fully convolutional encoder-decoder network was constructed for this task (Fig. 5), which takes single-channel grayscale images as input. The encoder contains two convolutional layers, with the first layer—implemented on our chip—varying in kernel size (3 × 3, 5 × 5, 7 × 7) to simulate different receptive fields. The decoder uses a symmetric structure to reconstruct the denoised image. All convolutional layers are padded to maintain spatial dimensions.

**Figure 5.**
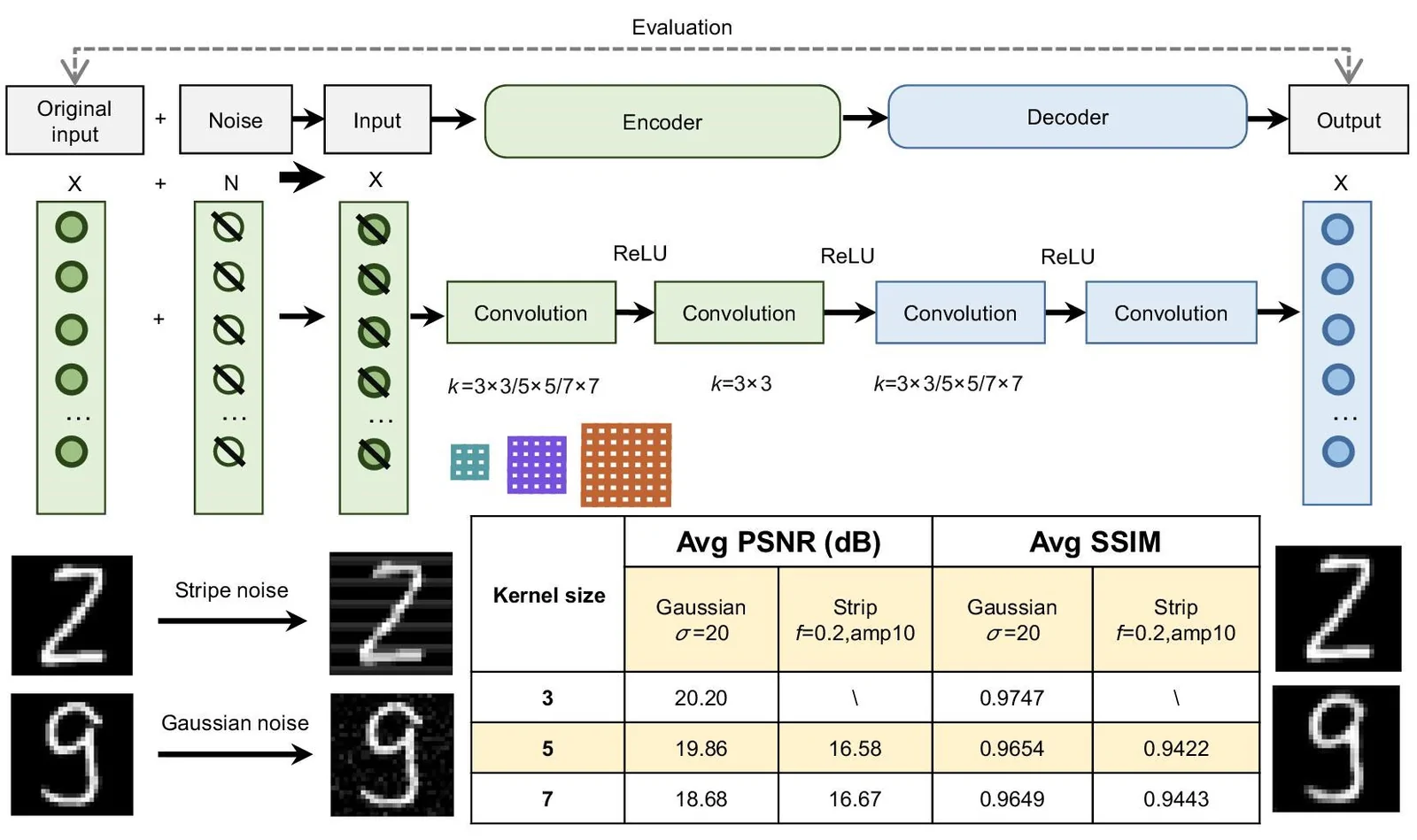
Schematic of the denoising neural network architecture and the denoising performance evaluation of networks with varying convolutional kernel sizes.

Experiments were performed on 500 images from the EMNIST dataset, split into 400 training and 100 validation samples. The network was evaluated under two types of noise: Gaussian noise (*σ* = 20) and stripe noise (*f* = 0.2, *a* = 10), with performance measured by peak signal-to-noise ratio (PSNR) and structural similarity index measure (SSIM). As summarized in Fig. 5, smaller kernels performed better under Gaussian noise, with the 3 × 3 kernel achieving the highest PSNR (20.20 dB) and SSIM (0.9747). This is attributed to its strong local feature extraction capability, which effectively filters spatially random noise. In contrast, larger kernels led to detail oversmoothing and reduced accuracy. For structured stripe noise, the 7 × 7 kernel performed best (PSNR 16.67 dB, SSIM 0.9443), as its broader receptive field helps integrate contextual information to suppress periodic interference. Because the publicly available EMNIST images have a low resolution (28 × 28), the performance gains from large convolution kernels are more pronounced when processing complex scenes.

These results demonstrate that the kernel size critically influences denoising performance and should be chosen according to noise characteristics. Small kernels excel at removing local random noise, while large kernels perform better to handle structured noise through global context integration. This underscores the value of reconfigurable multiscale kernel support in both hardware and algorithm design for adaptive visual processing.

## DISCUSSION AND CONCLUSION

In this work, we propose a multidimensional multiplexed optical matrix–multiplication chip that leverages wavelength, mode, and polarization to fully exploit the DoFs of light, achieving 192 parallel computing channels. This high degree of parallelism enables both large and flexibly reconfigurable convolution kernels, significantly enhancing processing throughput and computational density. Experimental results demonstrate that the proposed architecture supports flexible convolution operations with kernel sizes up to 13 × 13, enabling multiscale feature extraction and diverse image processing tasks. We compared the number of optical DoFs utilized and the relevant performance metrics in several representative studies, as summarized in Table 1 [20,29,40–44]. It can be observed that prior works have demonstrated the theoretical potential to support up to 75 or even 100 parallel channels via WDM [41,42]. However, the available optical DoFs have not been fully exploited, as most studies rely solely on the wavelength dimension. In contrast, a number of works have explored additional dimensions. Our group has introduced the mode dimension into optical computing architectures, using four modes to achieve four parallel channels [44]. Lanzhou University employed three spatial modes and four wavelengths to achieve 12 parallel channels [29]. Nevertheless, the scale of these demonstrations remains relatively limited and does not fully reveal the potential of multidimensional multiplexing for substantially increasing parallelism. To the best of our knowledge, the present work is the first to realize highly parallel on-chip optical computing by simultaneously exploiting wavelength, mode, and polarization dimensions. We further validated the system’s reconfigurability by performing image processing using kernels of various sizes and by implementing a comparative denoising neural network on-chip, confirming its practical effectiveness across diverse vision tasks. The optical chip can achieve a throughput of 15.36 TOPS, which can be further enhanced through higher-speed modulation and expanded beyond optical dimensionality [20] to support more demanding applications. Further discussion on large-scale systems and scalability can be found in Note S5.

**Table 1.** Summary of performance in multi-dimensional parallel photonic computing chips.

Year [ref.]	No. of wavelengths (λ)	No. of modes	No. of polarizations	Total parallel optical dimensions	No. of dimensions in the actual demonstration	Maximum throughout using the chip (TOPS)	On chip power consumption (TOPS/W)
2026 (this work)	32	3	2	192	49	15.36@40 Gbaud (3.92@40 Gbaud in experiment)	27.98
2025 [40]	>4	0	0	>4	4 (×3 in RF domain)	0.245@30.67Gbaud	0.267
2025 [41]	75	0	0	75	3	37.5TOPS@10Gbaud	/
2025 [42]	100	0	0	100	1	2560TOPS@50Gbaud	3.2
2025 [43]	>2	3	0	>6	6	52.56@4.38/2K Tbaud (K: number of wavelengths)	/
2023 [29]	4	3	0	12	12	*/*	*/*
2023 [44]	0	4	0	4	4	*/*	*/*
2023 [20]	>6	0	0	>6	6 (×50 in RF domain)	*/*	*/*

Although this work represents a substantial advancement in the programmability and adaptability of optical convolutional processors, further research is necessary to realize on-chip nonlinear activation, improve the reconfiguration accuracy of multikernel setups, and develop co-optimized photonic-electronic training algorithms [45–50]. Overall, the proposed architecture provides a promising pathway toward high-efficiency, low-latency, and highly generalizable optoelectronic computing systems, particularly suited for edge computing and real-time vision applications.

## METHODS

### Chip fabrication method

The proposed WMP-multiplexed matrix multiplication chip was fabricated on a standard silicon-on-insulator (SOI) substrate, comprising a 220-nm-thick top silicon layer and a 3-μm-thick buried oxide layer. The silicon photonic waveguides were patterned via electron-beam lithography and subsequently dry-etched. A 1.5 μm-thick SiO₂ upper cladding was then deposited via plasma-enhanced chemical vapor deposition. For thermal tuning, microheaters composed of Cr (20 nm)/Ti (300 nm) were fabricated, while electrical interconnects were formed using Cr (30 nm)/Au (300 nm) stacks, optimized for the coupled local-intrinsic plasmonic polariton (CLIPP) structures. A 200 nm-thick SiO₂ passivation layer was deposited to protect the metal layers, with contact windows opened by dry etching.

### Measurement details

The input signals, generated by an AWG (Keysight M8194A) operating at 120 GSa/s with root-raised cosine filter shaping, were fed into a modulator (iXblue MXAN-LN-40). Multiple tunable lasers (LBTEK TLS1056) served as the light sources, with their input polarization adjusted using polarization controllers (JCOPTIX FAP-C56). The fully packaged chip was mounted on a temperature-controlled platform and stabilized at 25°C. All tuning elements were thermally reconfigured via microheaters driven by a programmable control circuit (T-MS160-12CV PCC). Following chip processing, the signal was amplified by an erbium-doped fiber amplifier (Amoincs AEDFA-PA-35-B-FA) to compensate for on-chip insertion loss. The output from different channels was detected separately by a high-speed real-time oscilloscope (Keysight DSOZ594A). The AWG and oscilloscope were synchronized to ensure stable waveform acquisition, with the oscilloscope sampling at 80 GSa/s and the data subsequently down-sampled to 40 GSa/s for result reconstruction. A central computer coordinated the setup by uploading waveform files to the AWG.

## Supplementary Material

nwag350_Supplemental_File
