# Supplementary material for: 192-dimensional wavelength-/mode-/polarization-multiplexed photonic architecture for highly parallel optical computing
Source: Natl Sci Rev. 2026 Jun 8;13(19):nwag350. doi: 10.1093/nsr/nwag350 (PMC13637628; doi:10.1093/nsr/nwag350)
Supplement: Supplementary file 1 [file nwag350_supplemental_file.pdf]

Supplementary Information for:

A 192-dimensional wavelength-/mode-/polarization-multiplexed photonic architecture for highly-parallel optical computing

Qingrui Yao[1,2], Yuxin Sun[1], Weike Zhao[1], Yiwei Xie[1], Yingying Peng[1], Huan Li[1], Pan Wang[1], Xiangxing Bai[2], Yaocheng Shi [1,3,4], and Daoxin Dai [1,3,4]

1. State Key Laboratory for Extreme Photonics and Instrumentation, College of Optical Science and Engineering, International Research Center for Advanced Photonics, Zhejiang University, Zijingang Campus, Hangzhou 310058, China.
2. QIANYUAN Laboratory, Hangzhou 310058, China.
3. Intelligent Optics and Photonics Research Center, Jiaxing Research Institute, Zhejiang University, Jiaxing 314000, China.
4. Ningbo Research Institute, Zhejiang University, Ningbo 315100, China.

*Corresponding author: xieyiw@zju.edu.cn; dxdai@zju.edu.cn.

Contents

S1. Polarization Control and Dual-Polarization Input Methods

S2. Additional information for design and characterization of the key building blocks

S2.1. Design and usage parameters of the mode/polarization (de)multiplexers

S2.2. Design and usage parameters of the EMRs

S3. Image Preprocessing and Operational Procedures

S4. Estimation of throughput, computing density, and energy consumption

S5. Discussion on large-scale systems and scalability

S1. Polarization Control and Dual-Polarization Input Methods

The proposed wavelength-/mode-/polarization (WMP)-multiplexed photonic chip employs a dual-polarization input scheme. A schematic diagram of the input ports, along with their respective labels, is provided in Fig. S1. Ports 1–3 serve as the main input ports of the processor, while ports 4–5 are U-shaped auxiliary ports mainly used for packaging. The dual-polarization modulated signals (each with 32 wavelengths) are injected into the chip through ports 1-3, each carrying both TE_0 and TM_0 polarization states. These inputs are subsequently converted by the on-chip mode/polarization multiplexers into TE_2/TM_2, TE_0/TM_0, and TE_1/TM_1 modes, respectively, and combined to transmit into the multi-mode bus waveguide (MBW).

Since the input light inherently contains both TE and TM modes, we only need to control their powe ratio to 1:1. Polarization control is achieved using a polarization controller (PC) within the system. To minimize the impact of environmental disturbances, polarization-maintaining fibers are utilized in the setup.

In the experimental system, the polarization state is adjusted with the PC and set to 45° linear polarization using a polarimeter. Once this state is established, all optical fibers in the system are secured to maintain stability, and the polarization-maintaining fiber connected to the chip is then engaged.

During the experiments, the on-chip mode control remained relatively stable. This is primarily because the individual components, such as the mode and polarization multiplexing devices, do not require thermal tuning, and exhibit stable performance. Additionally, the system's input and output are equipped with polarization controllers to maintain polarization control and ensure system stability. During long-term operation, no noticeable issues such as mode-channel crosstalk have been observed.

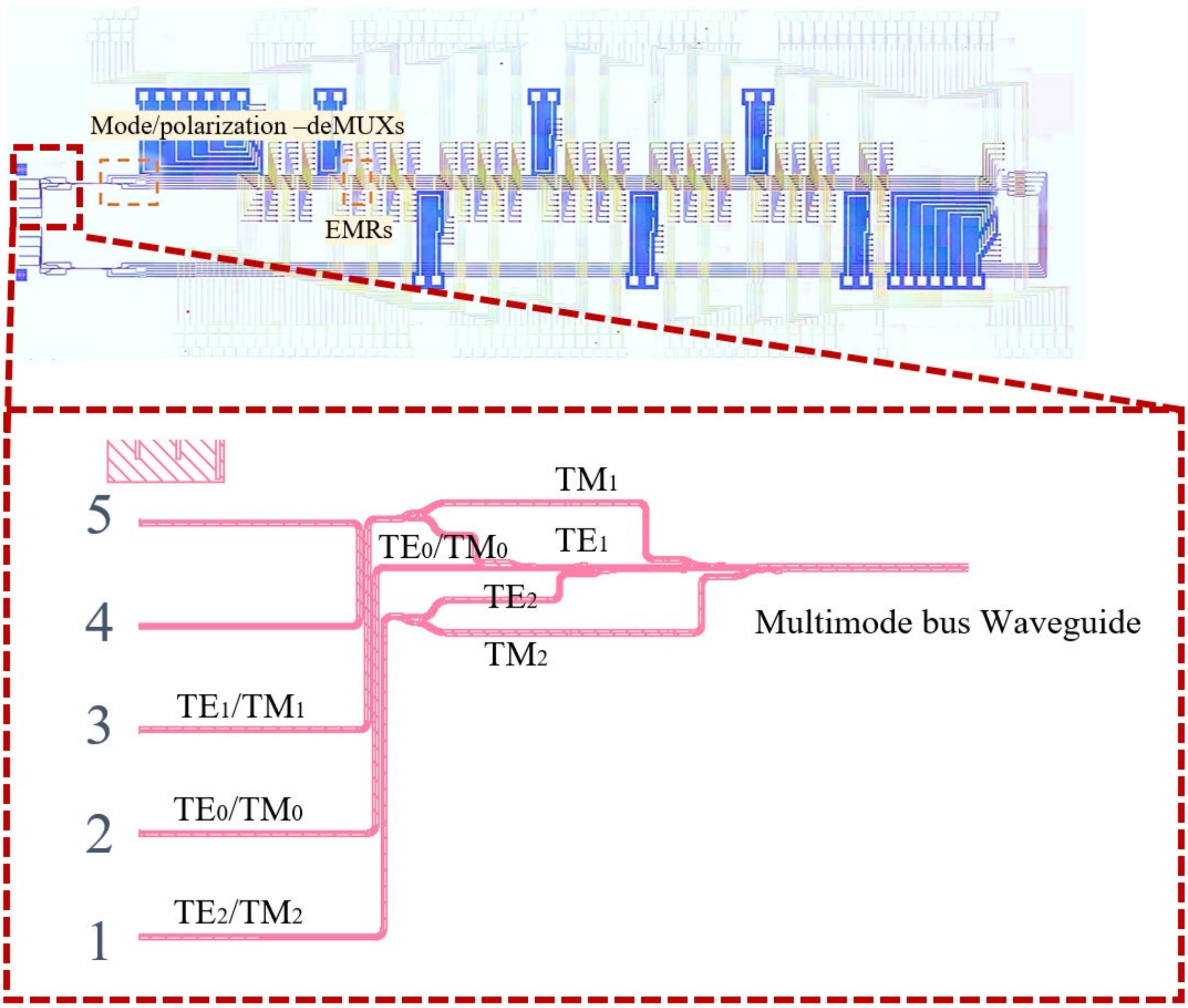

Figure S1 | Schematic of the dual-polarization input ports for the wavelength-, mode-, and polarization-(WMP) multiplexed photonic chip.

S2. Additional information for design and characterization of the key building blocks

S2.1. Design and usage parameters of the mode/polarization (de)multiplexers

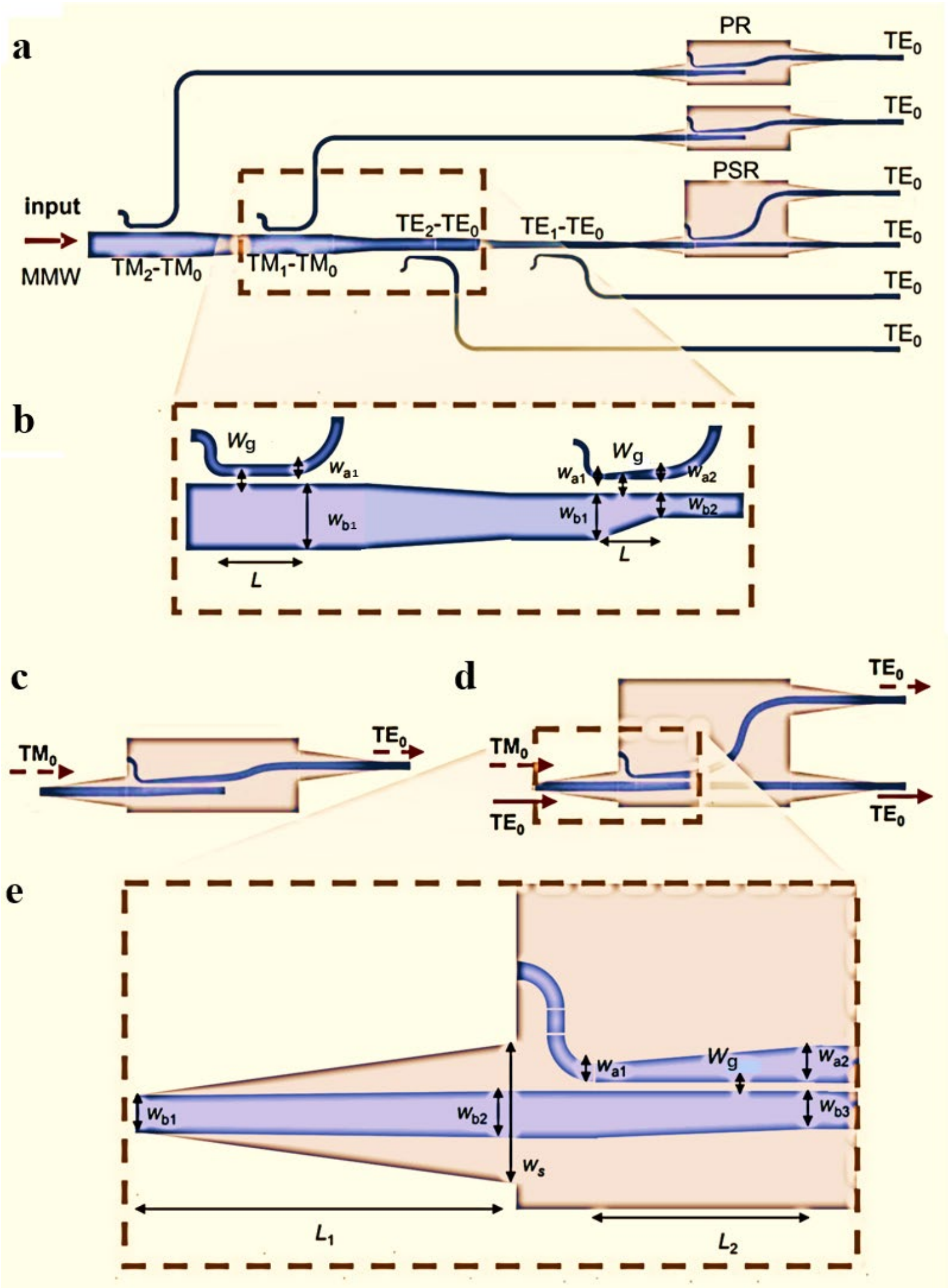

Figure S2 | Schematic diagram of the mode/polarization (de)multiplexer structures. a Architecture of the six-channel demultiplexer for mode/polarization (de)multiplexer. **b** Geometrical parameters of the multiplexers for TE and TM modes. **c** PR; **d** PSR; **e** The parameter setings of PSR.

Figure S2 (a) presents a schematic of the six-channel mode/polarization (de)multiplexer. In the MBW, higher-order modes (TM_2, TM_1, TE_2, and TE_1) are sequentially demultiplexed into TM_0 and TE_0 modes. The resulting TM_0 modes are subsequently converted into TE_0 modes via dedicated polarization rotators (PRs). Meanwhile, the remaining TM_0 mode propagating in the MBW is processed by a polarization splitter-rotator (PSR), which converts it into a TE_0 mode output at the cross port. The TE_0 mode traveling in the MBW remains unaltered and is directed through the through port of the PSR. Figure S2 (b) illustrates the structural layouts and

parameter configurations of the TM- and TE-mode multiplexers, respectively, with all parameter values adopted from our previous work. Detailed values for these parameters are provided in Table S1 [1-3].

The operation of the PSR leverages the vertical asymmetry of the ridge waveguide, which induces mode hybridization between the TM_0 and TE_1 modes. Schematic diagrams of the PR and the PSR are presented in Fig. S2(c-d), respectively. As illustrated in Fig. S2(e), both device function on an identical working principle and share key structural parameters in their core regions, with a slab layer thickness of 70 nm. Other key parameters are summarized in Table S2.

Table S1. Parameters of the designed couplers for four higher order modes.

**Parameters (μm)**	w_{b1}	w_{b2}	w_{a1}	w_{a2}	L	w_g
**TM_2**		1.695		0.4	5.7	0.3
**TM_1**		1.092		0.43	5	0.3
**TE_2**	1.04	0.93	0.26	0.32	50	0.18
**TE_1**	0.62	0.44	0.2	0.29	40	0.18

Table S2. Parameters of the designed PSR.

**Parameters**	w_{b1}	w_{b2}	w_{b3}	w_s	w_{a1}	w_{a2}	w_g	L_1	L_2
**(μm)**	0.42	0.72	0.42	2	0.18	0.42	0.18	130	60

S2.2. Design and usage parameters of the EMRs

Figure S3(a) schematically illustrates the structure of our elliptical microring resonator (EMR). The EMR structure provides greater resonant freedom by adjusting the ratio of the major to minor axes, thereby optimizing the resonance spectrum. This design features a compact structure, effectively increases the free spectral range (FSR), and supports a broader range of wavelength selection. Compared with conventional circular microrings, elliptical microrings are less sensitive to fabrication errors and offer greater tolerance. They exhibit stronger consistency in large-scale fabrication, making them more advantageous for applications in large-scale systems. A fixed radius difference (ΔR) between successive EMRs is employed to achieve a channel spacing of 100 GHz (0.8 nm). Although the fabricated arrayed EMRs exhibit slight deviations in their central wavelengths from the designed values, this equidistant approach still helps reduce the tuning power to some extent. A smaller bending radius naturally ensures a broader FSR for the EMR, thereby accommodating more wavelength channels.

The EMR is implemented using adiabatically tapered elliptical waveguide design: in the non-coupling region, the core width W_a is widened to allow a minimal bending radius R_a, whereas in the coupling region, the core width W_b is narrowed to achieve sufficient coupling. Furthermore, the bus waveguide width W_{bus} and bending radius R_b are optimized to significantly reduce mode-mismatch loss in the coupling region. The corresponding parameters are summarized in Table S3, where K represents the coupling coefficient.

Figure S3(b) presents the simulated spectral response at the through port of the 32 EMRs. The results indicate an FSR of approximately 30 nm and a channel spacing of about 100 GHz, enabling support for 32 wavelength channels with a simulated loss of around 0.1 dB.

The Microscope image of EMR is shown in Fig. S3(c). The EMR has a measured resistance of approximately 350 Ω in the experiment, and effective tuning can be achieved with a voltage ranging from 0 to 1 V, which poses virtually no risk of device damage at this power level. To mitigate this possible thermal crosstalk effect, air trenches were introduced between adjacent devices to improve thermal isolation. In addition, a thermoelectric cooler (TEC) was installed beneath the chip to provide temperature stabilization during operation, further reducing thermal interference. As a result, the overall system can operate under stable conditions.

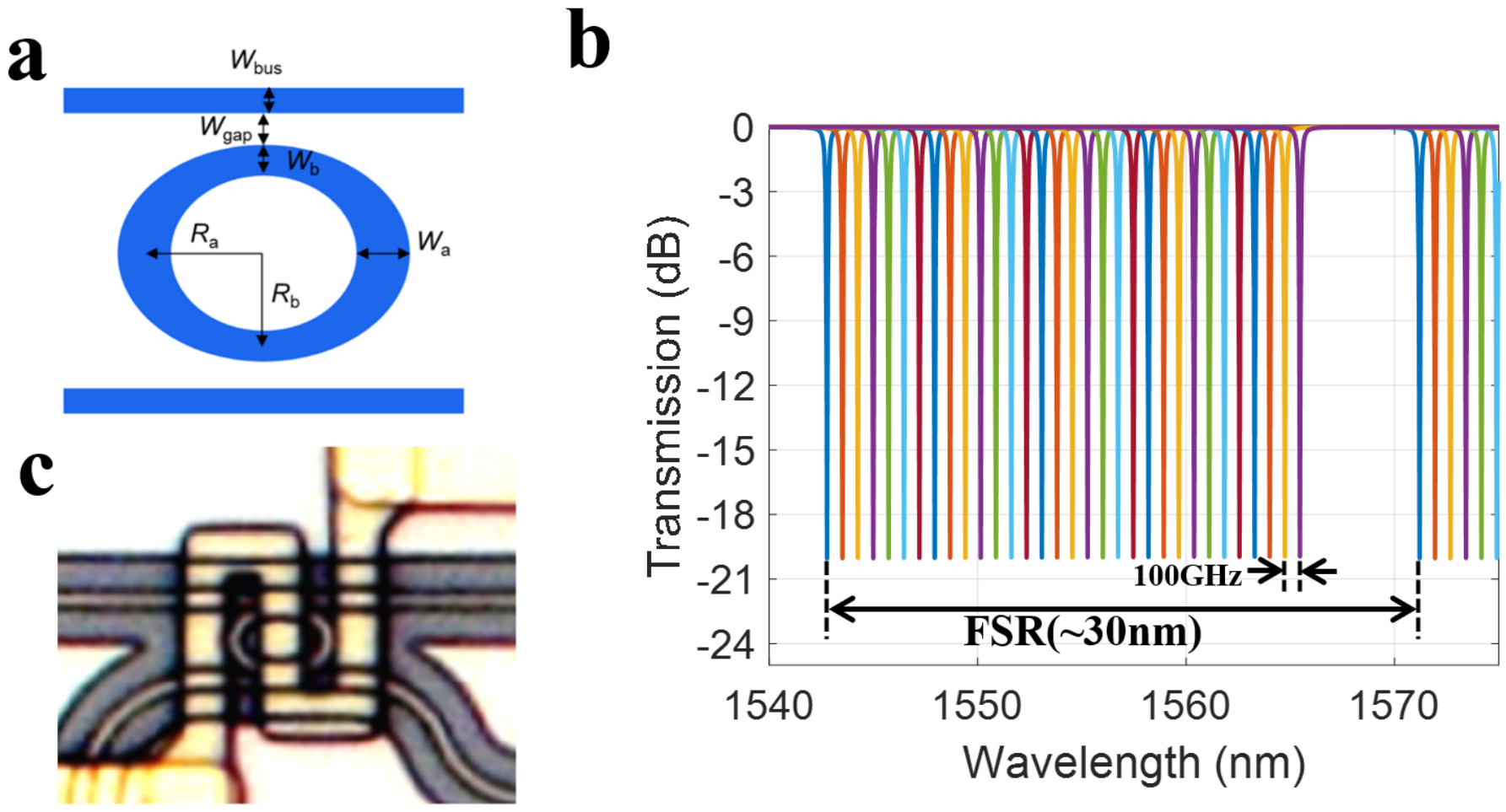

Figure S3 | Simulation and design of the EMR. **a** Schematic of the EMR structure. **b** Simulated spectral response of the EMRs.**c** Microscope image of EMR. **c** The image of the fabricated EMR.

Table S3. Parameters of the designed EMRs

**Parameters**	Ra	Rb	W_a	W_b	W_{bus}	W_{gap}	ΔR	FSR
**µm**	3.8	2.6	0.65	0.4	0.4	0.262	2.74×10^{-3}	~30nm

S3. Image Preprocessing and Operational Procedures

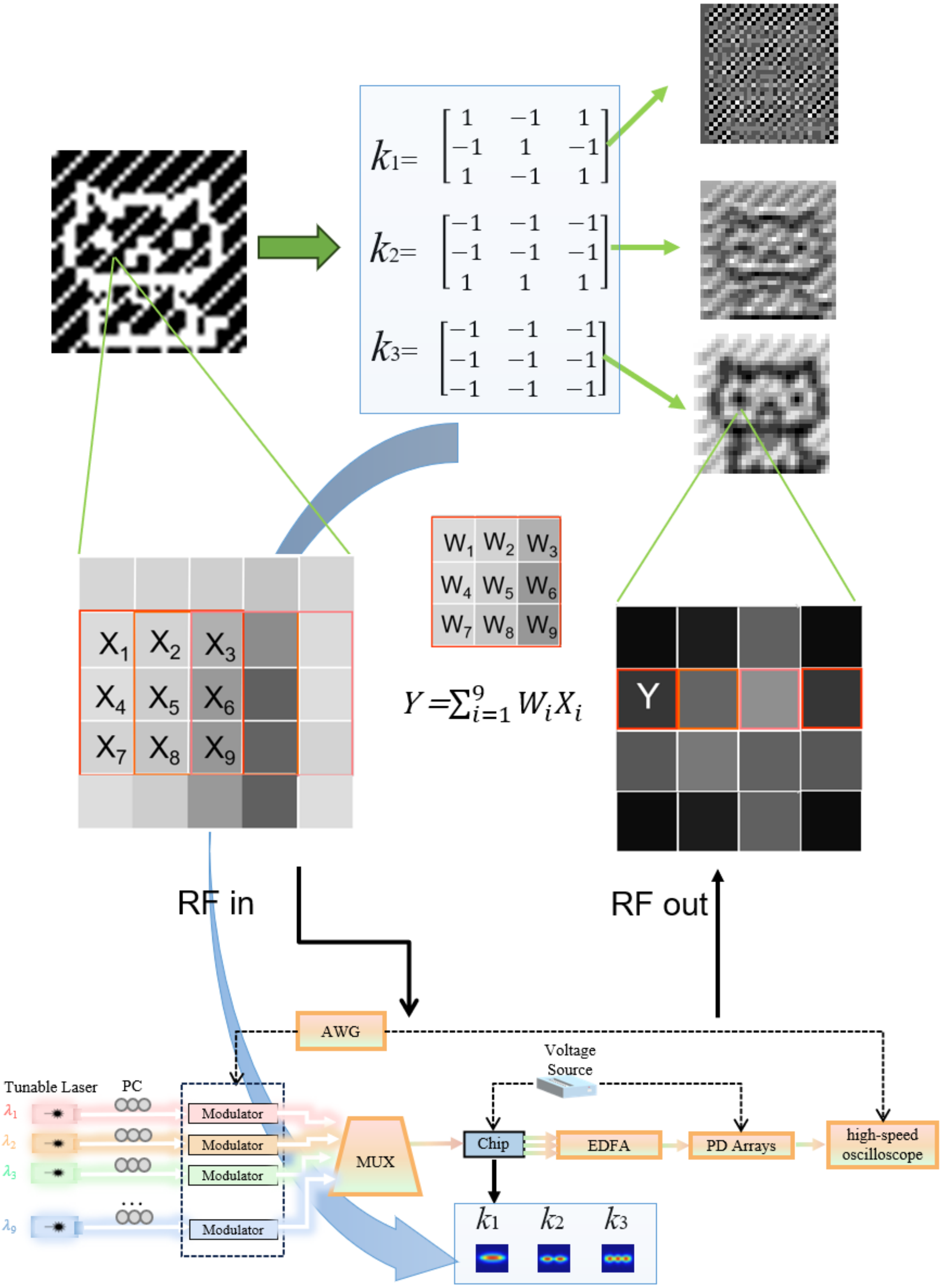

Figure S4 | Schematic of the convolution operation and experimental setup for the parallel image processing using three distinct 3×3 kernels (k1-k3).

The implementation of convolutional image processing and image preprocessing are well-established [4-6]. Here, we describe the data processing and experimental methodology used in our study, which involves two parallel convolution schemes: applying multiple 3×3 kernels with distinct physical meanings to the same image, and processing the image with kernels of different sizes. The schematic diagrams of these convolution operations, along with the resulting output images and the corresponding experimental setup, are presented in Fig. S4 and S5.

When three parallel processes are applied to the same input signal, different spatial mode channels are employed to process the signal simultaneously. Distinct wavelength channels are independently modulated with the corresponding input data and then coupled into the chip via a wavelength-division multiplexer. Figure S4 illustrates the data processing workflow and experimental setup.

Furthermore, to better compare the effects of different convolution kernel sizes, convolution operations were performed on the same image using three kernels of different sizes. To enable a fair comparison and ensure identical output dimensions, padding was applied to the input data. Accordingly, although the same image is processed, the modulation signals for the parallel input channels require are different. Figure S5 illustrates the data processing workflow and experimental setup for these parallel convolutions using different-sized kernels (k_4-k_6) on the variably padded inputs.

The detailed process of how we utilize the EMR to achieve weight tuning is as follows. We first unify the convolution kernel parameters required for a single computation within the range of 0 to 1. The two states we demonstrate, 0 V and 1 V, represent the extreme states of the EMR switch. We treat the optical intensities, i.e., the device weights, at these two voltages as the '0' and '1' states. By sweeping this voltage interval, we obtain the relationship between the voltage and the device weight, covering all intermediate states. Finally, we map the normalized convolution kernel parameters to the device weights, and subsequently to the tuning voltages required for the device. As shown in Fig. 2f, the extinction ratio between the '0' and '1' states exceeds 20 dB. Additionally, as shown in Fig. 2a, our system features numerous test ports that facilitate monitoring the status of each individual device.

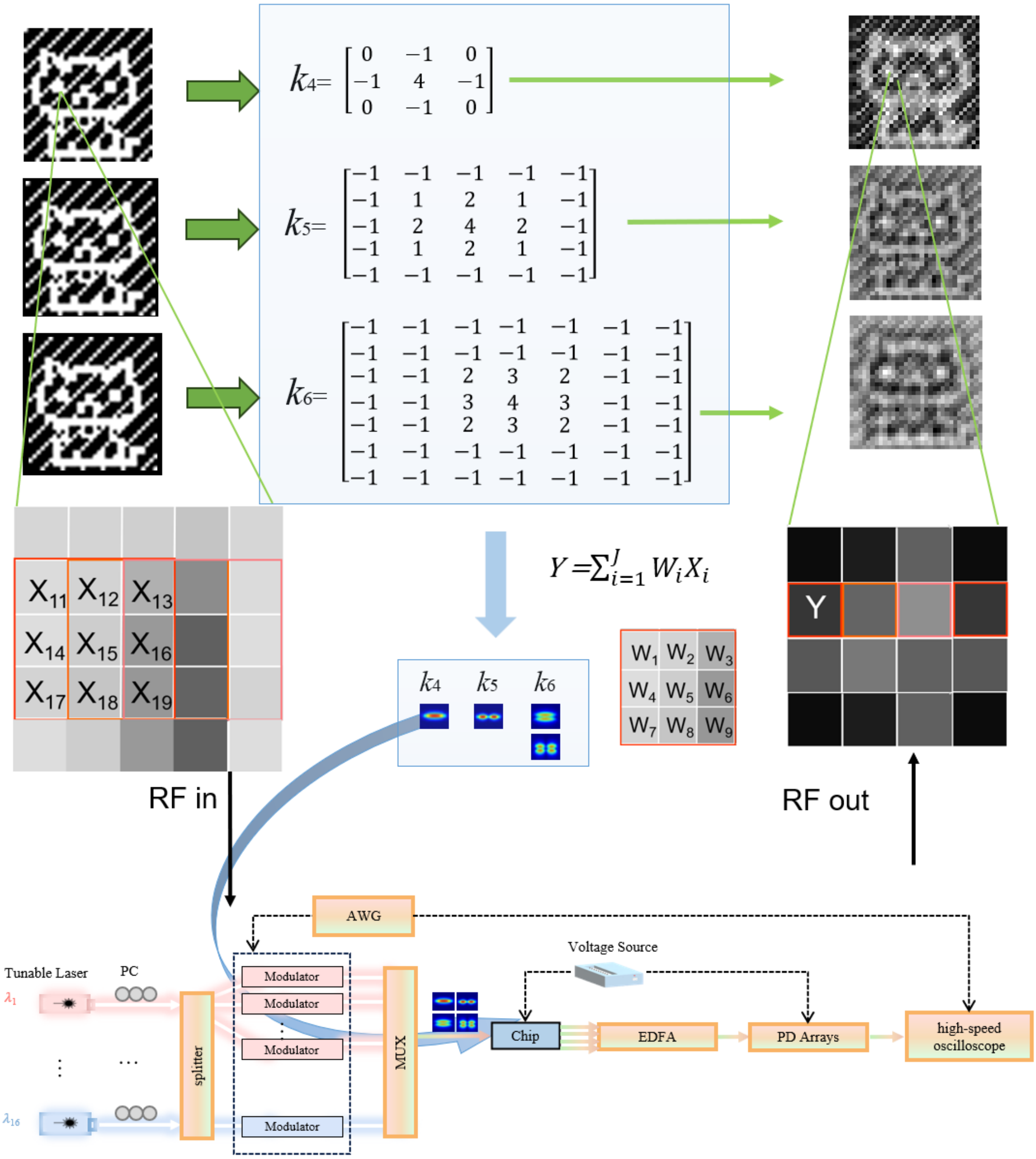

Figure S5 | Schematic and experimental setup for parallel image processing using three different-sized convolutional kernels (k_4–k_6).

S4. Estimation of throughput, computing density, and energy consumption

In computing hardware evaluation, three principal metrics govern performance assessment: throughput, computational density, and energy efficiency [7]. Furthermore, when evaluating the computational capability, we consider the potential computational power that this structure can theoretically achieve under the assumption that the chip is fully packaged and there is a sufficient number of external devices to modulate all the wavelengths, such as input signal equipment.

Throughput: For high-performance computing systems, throughput is commonly quantified in operations per second (OPS), representing a processor's fundamental computational capacity. In photonic computing architectures, the throughput (T) can be expressed as:

$$T = 2m \times N \times r \text{ OPS} \tag{S1}$$

The throughput (T) in OPS represents the computational capacity of the photonic architecture, where T excludes off-chip data loading overhead and is determined by several key parameters: Since the convolution process involves both multiplication and addition, each convolution operation is counted as two arithmetic operations. m denotes the number of parallel photonic computing layers, equivalent to the available mode/polarization channels; N corresponds to the dimension of the on-chip weight matrix; while r characterizes the operational rate governed by the synchronization between signal modulation efficiency and photodetector response.

Our WMP multiplexed photonic chip inherently implements multiply-accumulate (MAC) operations, with each MAC comprising both multiplication and accumulation phases. Accordingly, our throughput calculation counts each MAC as two fundamental operations. Leveraging our photonic chip, the system can perform six parallel 32 multiply operations simultaneously, completing 192 multiplication operations per cycle. The modulation rate we used in the experiment was 40 Gbaud, shaped with an root-raised cosine (RRC) filter. Under these conditions, the computational speed of the chip reaches15.36 TOPS, calculated as 2× 6 × 32 × 40 Gbaud. Furthermore, the computational performance of the chip can be further enhanced by employing modulators with higher speeds, increasing the number of parallel channels via a greater number of modes/polarizations, or utilizing microring resonators with a larger FSR for a increased number of wavelength channels.

Computational density: Following the throughput characterization, we evaluate the computational density (C) of the MVM architecture, representing the operational capacity normalized by chip area, through Equation (S2):

$$C = T/F \tag{S2}$$

where C represents the computing density, and F is the footprint of the photonic computing core. The computational density of this optical computing system is 0.85 TOPS/mm^2 , based on the core optical devices area of 13.9 mm × 1.3 mm (excluding the edge couplers and electrical electrodes). Since the chip is fabricated in-house, its layout is spacious with widely distributed electrodes, allowing the computational density to be increased by several times with ease.

Power consumption: The power consumption of chip includes integrated chips and external desktop instruments. The power consumption of our WMP multiplexed photonic chip primarily stems from just one key components: EMR state configuration. For the experimental

prototype employing 32 wavelengths, we evaluate the worst-case power scenario by assuming all devices operate at their maximum power consumption states.

Each EMR requires a maximum tuning voltage of 1 V to control the signal amplitude from 0 to 1 and a resistance of 350 Ω. Thus, the entire on-chip computational pipeline consumes merely 0.549 W at peak operation and correspondingly the on-chip power efficiency is 27.98 TOPS/W. Definitely, peripheral elements also consume some additional power. The external elements include multi-wavelength lasers, modulators, photodetector and thermoelectric coolers (TEC). To streamline the system, an optical frequency comb could be adopted to reduce the device count and complexity [8-10]. To fully exploit all wavelength channels, an optical frequency comb is employed as the light source. The total power consumption for a typical comb source is approximately 4.75 W, which includes the temperature control unit and the devices related to laser generation. The power required by each modulators is 0.5 W, and the number needed varies depending on the specific functionality to be implemented. The six detectors together require 0.6 W. The total power consumption can be improved potentially by integrating all components such as modulators and detectors onto a single chip. In contrast, the traditional electronic computing systems consume over 350 W [11-12] including the power from on-chip memory, I/O operations, control logic and so on.

Since data loading and weight reconfiguration can become practical bottlenecks for the net computing throughput of photonic computing systems, these factors should also be considered. In our architecture, multiple input data elements are simultaneously injected into the AWG and encoded onto distinct wavelengths and orthogonal spatial modes using independent electro-optic modulators. This process introduces a short but fixed AWG data-loading and response time. Once loaded, the data are continuously streamed into the chip at the preset modulation rate (e.g., 40 Gbaud). Additional latency from electrical interconnects and optical fiber links may also exist, but these delays do not affect the intrinsic computing speed and can be reduced by shortening the connection lengths. Importantly, as the convolution kernel size (i.e., chip scale) and the amount of input data increase, the AWG loading time and interconnection delays remain nearly unchanged, while the computational throughput scales proportionally with the computation size. For weight reconfiguration, the convolution kernel weights are programmed through thermo-optic tuning of the EMRs using integrated heaters, with a response time on the order of microseconds. For applications requiring real-time weight updates, faster tuning mechanisms, such as electro-optic phase shifters, could be adopted in future implementations.

S5. Discussion on large-scale systems and scalability

We further discuss here the main limiting factors that may constrain further dimensional expansion. The current system design supports 192 parallel channels. We believe that further improving the degree of parallelism mainly involves two considerations. The first is to further increase the capacity of each existing dimension, such as the number of wavelength channels used in wavelength-division multiplexing and the number of modes used in mode-division multiplexing. In the current system, only 32 wavelengths are used. This number could be further increased by incorporating additional interleavers to increase the channel number and employing cross-FSR multiplexing techniques [13]. Similarly, increasing the number of spatial modes can also enhance parallelism, although higher-order modes typically suffer from larger propagation losses [14]. Second, further scalability may require incorporating additional dimensions, such as the radio-frequency (RF) domain [15], to achieve further expansion.

Although scaling inevitably introduces increased loss and system complexity, these challenges are manageable. Thanks to the ultra-low loss broadened waveguide and compact size of the EMRs, they exhibit relatively low loss at off-resonant wavelengths. As a result, scaling in the wavelength domain does not introduce excessive additional loss. In addition, crosstalk can be suppressed below –15 dB through optimized mode (de)multiplexer design, while thermal crosstalk in large-scale integration can be effectively mitigated using air trenches and thermoelectric cooling to ensure stable long-term operation. Meanwhile, the compact footprint of EMRs and mode (de)multiplexers minimizes area overhead during scaling, and the increased control complexity from additional electrodes can be alleviated by incorporating test ports for efficient calibration and state characterization.

Due to the large scale and high complexity of our structure, additional attention needs to be paid to certain issues during actual experiments. In practice, multi-wavelength input also requires special attention to ensure that the total power does not exceed the level that may induce effects such as two-photon absorption. The input power of the lasers must be precisely controlled: it should be high enough to maintain detectability, yet low enough to avoid nonlinear effects like two-photon absorption. To address this challenge, the maximum tolerable total power within a channel should first be determined. Then, based on this value, the optimal per-wavelength power can be selected to achieve higher power in each channel, thereby obtaining a better extinction ratio and signal-to-noise ratio.

However, system complexity can also be reduced through other approaches, such as integration with optical frequency combs to replace tunable lasers [8-10]. Currently, the technology and applications of optical frequency combs have become increasingly mature, offering a sufficiently large number of comb lines for practical use while maintaining low power consumption. Integrating optical frequency combs can not only significantly reduce system complexity but also lower energy consumption, while simultaneously facilitating further miniaturization of the overall system.

References

1. Zhao W, Peng Y, Cao X et al. 96-channel on-chip reconfigurable optical add-drop multiplexer for multidimensional multiplexing systems. *Nanophotonics* 2022; **11**: 4299-313.
2. Liu D, Zhang L, Tan Y and Dai D. High-order adiabatic elliptical-microring filter with an ultra-large free-spectral-range. *J Lightwave Technol* 2021; **39**: 5910-6.
3. Peng Y, Zhao W, Yi X et al. High-order adiabatic elliptical-microring filter with an ultra-large free-spectral-range. *ACS Photonics* 2025; **12**: 4897-906.
4. Yao Q, Sun Y, Ke X et al. Parallel matrix-vector multiplication using a scalable multiport interferometer. *Laser Photonics Rev* 2025; e01876.
5. Yin R, Xiao H, Jiang Y et al. Integrated WDM-compatible optical mode division multiplexing neural network accelerator. *Optica* 2023; **10**: 1709-18.
6. Huang Y, Huang B, Cheng C et al. Feature extraction from images using integrated photonic convolutional kernel. *IEEE Photon J* 2022; **14**: 8829207.
7. Cheng J, Huang C, Zhang J et al. Multimodal deep learning using on-chip diffractive optics with in situ training capability. *Nat Commun* 2024; **15**: 6189.
8. Feldmann J, Youngblood N, Karpov M et al. Parallel convolutional processing using an integrated photonic tensor core. *Nature* 2021; **589**: 52-8.
9. Zhou Z, Ou X, Fang Y et al. Prospects and applications of on-chip lasers. *eLight* 2023; **3**: 1.
10. Bai B, Yang Q, Shu H et al. Microcomb-based integrated photonic processing unit. *Nat Commun* 2023; **14**: 66.
11. NVIDIA. NVIDIA H100 Tensor Core GPU Datasheet. NVIDIA, 2024.
12. NVIDIA. NVIDIA A100 Tensor Core GPU. NVIDIA.
13. Rizzo A, Novick A, Gopal V et al. Massively scalable Kerr comb-driven silicon photonic link. *Nat Photonics* 2023; **17**: 781-90.
14. He Y, Zhang Y, Zhu Q et al. Silicon high-order mode (de)multiplexer on single polarization. *J Lightwave Technol* 2018; **36**: 5746-53.
15. Dong B et al. Higher-dimensional processing using a photonic tensor core with continuous-time data. *Nat Photonics* 2023; **17**: 1080-8.
